# Metal-Based Therapeutic Approaches for Overcoming Cancer Drug Resistance: Mechanisms, Drug Delivery Strategies, and Clinical Perspectives

**DOI:** 10.32604/or.2026.077445

**Published:** 2026-05-21

**Authors:** Kirill V. Chernov, Artemii M. Savin, Daria E. Otvodnikova, Oleg A. Kuchur, Sergey A. Tsymbal

**Affiliations:** 1 Center for Molecular and Biological Sciences, National Research University ITMO, Saint-Petersburg, Russia; 2 Laboratory of Bio- and Cheminformatics, School of Computer Science, Physics and Technology, HSE University, Saint-Petersburg, Russia

**Keywords:** Drug resistance, metal-based formulations, metal nanoparticles (MNPs), oxidative therapy, reactive oxygen species, drug conjugates

## Abstract

The formation of drug resistance poses the ultimate threat in modern oncology. Targeted therapy lacks versatility, while conventional therapy is famous for its side effects. However, for the new therapeutics to address the challenge of drug resistance, such compounds should combine properties of both modalities. In this review, we argue that metal-based therapeutics are paramount substances for achieving this goal. The unique physico-chemical properties and metabolism of these compounds, as well as metals themselves, allow to realize unique activities in normal and cancer cells, including precise targeting, non-apoptotic cell death, and disruption of critical signaling pathways. Despite all the advantages, the number of approved metal-based drugs remains relatively low. This review discusses the advantages of metal-based therapeutics in combating cancer drug resistance, starting from the role of metals in carcinogenesis and ending with the modern strategies for therapy, diagnostics and drug delivery, as well as issues that hamper the development of new substances.

## 1 Introduction

Extensive development of cancer therapy brought a great number of advances that enhanced outcomes of therapeutic interventions, increasing patients’ life expectancy [1]. While therapeutic strategies are balancing between treatment efficacy and patients’ quality of life, a new challenge appears on the scene. It is the formation of drug resistance, which causes more than 50% of relapses on average across all cancer types, especially in older patient cohorts [2,3].

Treatment of drug-resistant tumors requires extensive therapeutic influence capable of achieving an acceptable level of efficacy. Now, drug resistance is tackled through the development of various approaches, and in this article, we will try to show why metal-based agents could be one of the best and first options to consider. Metal-based drugs have several key characteristics: (1) they often possess multiple different activities preventing resistance formation through distinct mechanisms shown below; (2) modern formulations are able to achieve a high level of targeting, exploiting various tumor and intracellular properties; (3) metals allow the creation of organic scaffolds of particular structure unachievable in carbon-only compounds [4]. Despite advances in medicinal chemistry and our vast current knowledge about biochemical and biological properties of metals, we still have an insufficient number of approved metal-based drugs [5]. Among the major reasons are side effects and poor solubility in water [6]. With increasing requirements for patients’ quality of life and development of targeted and personal approaches, it is one of the primary considerations that should be revised before developing a new anti-cancer drug. According to patent submissions, there is also a significant gap between organic compounds and metal-based structures. And even distribution between various metal-based drugs is not equal, with platinum, ruthenium, and gold being the most used elements [7].

These contradictory trends, personal approach versus the formation of drug resistance, bring us to the conclusion that metal-based therapy is a good compromise and among the first options that should be considered at least on the last lines of treatment. The modern panel of anticancer drugs is filled with carbon-based organic structures [8,9] for obvious reasons, however, this should not be the obstacle for the development of new metal-based therapeutics, especially for the severe cases of drug resistance formation.

This review aims to show the increasing feasibility and versatility of metal-based formulations, especially in cases of drug resistance in cancer. We will explore basic metabolism of metals and their role in cancer, examine known, reliable and clinically used drugs as well as modern therapies that are under development or in clinical trials, and discuss approaches to enhance the effectiveness and safety of metal-based therapeutics.

## 2 Physiologically Relevant Metals and Their Role in Cancer Development and Drug Resistance Formation

The unique properties of metals allow them to perform various functions in the organism and inside the cells. The important role of many metals is to serve as a cofactor for the enzymes that operate and orchestrate the intracellular environment. The functions of metals can vary depending on the type of enzyme and the element, starting from biochemical reaction catalysis and ending with complex biological processes, like cell division, DNA replication, and cell migration, which would be impossible without them [10]. It is crucial to understand the particular place and function of each metal and each metal-containing factor, as well as metal metabolism as a whole, to successfully develop new therapeutic strategies, find new targets, and design molecular compounds with ultimate therapeutic efficiency against cancer cells.

Since cells contain many important metals that can influence carcinogenesis to varying degrees, it is necessary to classify them based on their physiological functions and involvement in therapy [6,11].

**Figure 1:**
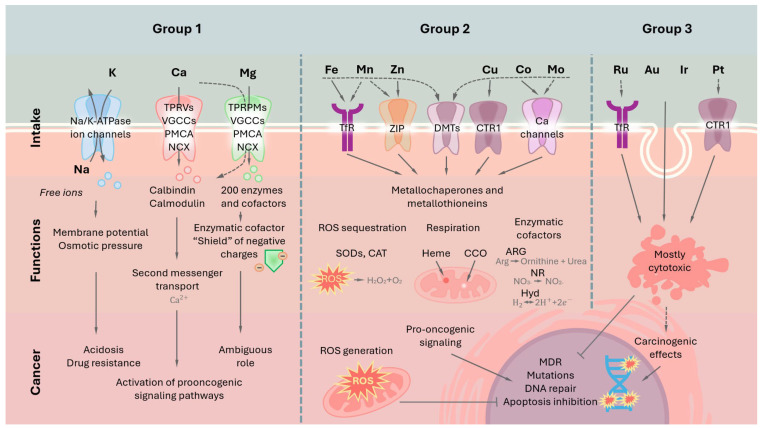
General scheme of metals’ role in healthy cells and cancer. Group 1 is represented by metals with high intraorganismal abundance. They perform critical functions in cell physiology as well as in tumor pathology. These metals interact with numerous protein factors, the expression of which often changes in cancer. Group 2 consists of transition metals that perform a set of specific functions in the cell. The expression profile of factors associated with the metabolism of these metals is often changed in cancer (see text for details). Finally, group 3 is composed of heavy metal elements that possess no known physiological function in the cell. They usually do not have any specific protein factors associated with their metabolism and can accumulate in the organism. Metals of these groups are either inert or can be profoundly toxic and carcinogenic.

Existing classifications of metals are based on their chemical and physical properties [12]. Herein, we will follow the general classification of mineral nutrients and divide metals into 3 groups: the first group (Group 1) of essential metals includes sodium, potassium, calcium, and magnesium. They occupy first positions in the list of metals according to their intracellular concentration [13] and mainly serve as buffer ions, important cofactors, or signaling factors. The second group (Group 2) includes trace elements like zinc, copper, iron, cobalt, and manganese, which are less abundant but play an important role in several biological processes and biochemical pathways. The third group (Group 3) is represented by the rest of the metals that can be met in the cell. The majority of them do not possess any known biological function, while some can be cytotoxic, or even carcinogenic, in relatively low concentrations (Fig. 1, Table 1). Further, we will dive deeper into the biological aspects of metals from each group with particular emphasis on carcinogenesis.

### 2.1 Group 1: Essential Biogenic Metals (K, Na, Ca, Mg)

**Figure 2:**
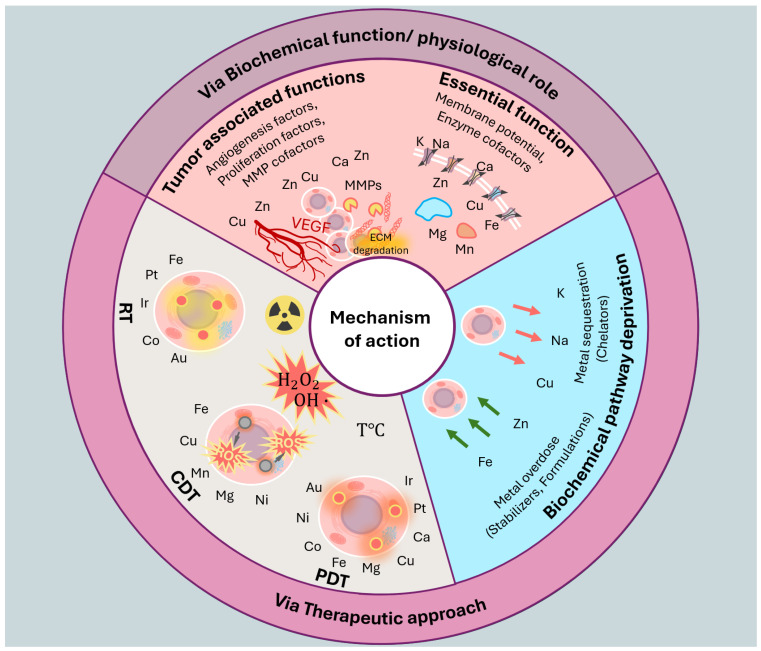
The mechanism of action of metal-based drugs can be divided into their therapeutic and physiological roles. In the former case, it is important to consider their essential (under normal conditions) and tumor-associated functions. In the case of therapeutic significance, we can consider both the deprivation of biochemical pathways essential for cell survival and the applied use of metals in therapy—radiation (RT), chemodynamic (CDT), and photodynamic therapy (PDT).

The first group of nutrient metals includes the most abundant of them—sodium, potassium, magnesium, and calcium. They function as regulators of osmotic pressure inside the cells and key regulators of biochemical processes (Fig. 1 and Fig. 2) [14]. 

The imbalance between intracellular and extracellular concentrations of sodium and potassium creates an electrochemical gradient or membrane potential that is a “vital force” for all living cells [15]. Membrane potential ensures the proper functioning of transmembrane transport enzymes, allowing passage of critical substances like glucose or Ca^2+^. High intracellular potassium concentration also facilitates the proper function of glycolysis, protein synthesis, and cell cycle progression [16].

The role of sodium and potassium in cancer was determined in the middle of the 20th century. While potassium promotes cancer from within the cells by inhibiting apoptosis, controlling the cell cycle, or changing cell shape, allowing metastasis [17,18], sodium acts in the extracellular environment, altering immune response [19] and the effectiveness of some weakly basic chemotherapeutic drugs like doxorubicin or vincristine promoting drug-resistant phenotypes of cancer cells. Prolonged treatment usually causes dysregulation of sodium or potassium metabolism in patients, resulting in hypernatremia or hypokalemia. These complications should be specifically addressed in order to avoid the development of more serious symptoms.

Calcium serves as a secondary messenger in numerous cellular signaling pathways [20]. The calcium release can trigger a variety of cellular responses, from changes in gene expression to metabolic shifts. The intake of the metal happens through numerous types of transporters, including non-selective ion channels, voltage-gated ion channels, and ligand-gated ion channels [21].

Magnesium is the most abundant divalent cation inside the cytoplasm. Its primary function is to serve as a cofactor for enzymes and “a shield” for negative charges on a variety of proteins. The absorption of Mg^2+^ is facilitated by 3 types of transporters: Mitochondrial RNA Splicing 2 (MRS2), Solute Carrier Family 41 (SLC41), and Transient Receptor Potential Melastatin 6/7 (TRPM6/7) transporters, which are hormonally regulated by insulin and epidermal growth factor. For intestinal intake, the intercellular transport conducted by claudin-2 and -12 is known. Some magnesium transporters can also transfer calcium ions through the membrane (but not *vice versa*) due to the similarities in hydrodynamic state and size. Magnesium is implicated in more than 600 enzymatic processes. It controls the proper work of ATP, mitochondria, the activity of vitamin D, and the elimination of oxidative stress [22]. 

These two metals play a key role in the formation, development and spread of cancer. Since calcium signaling is a crucial regulator of numerous processes involved in cell growth, some tumors (gliomas, breast, colorectal, prostate cancers, etc.) exhibit an increased expression of calcium transporters—TRPC, TRPM family, VGCCs [20]. This stimulates intracellular signaling that promotes activation of key pro-survival and growth factors (NFAT, NF-kB, c-Myc). These processes are well-characterized for prostate cancer, where calcium can promote cell survival and even participate in the formation of drug resistance [23]. The particular role of magnesium in cancer is still ambiguous. On the one hand, its increased consumption is linked to decreased risks of colon cancer in women, on the other—a positive correlation between high magnesium and breast cancer was also determined [24]. In a study on cultivated cancer cells, it was revealed that they indeed tend to accumulate more Mg^2+^ ions, which is explained by increased ATP demands [25]. However, *in vivo* studies indicate that low magnesium can simultaneously support and limit cancer burden [26]. So, for now, magnesium appears as a double-edged sword in cancer since it can either stimulate or inhibit its growth depending on the conditions and model used.

Thus, essential biogenic metals play a huge role in normal cell physiology and act as important factors in cancer development and progression. They can promote malignant cell growth or suppress it, depending on the conditions, which should be carefully considered by clinicians.

### 2.2 Group 2: Essential Trace Elements (Zn, Fe, Cu, Mn, Co, Ni, Mo)

Transition elements such as iron, copper, manganese, cobalt, molybdenum, nickel and post-transition metal zinc are important trace elements that play diverse roles in the cell, being catalytic and, mostly for Zn, structural co-factors [27]. Fe and Cu are typical redox-active metals, which participate in Fenton-like reactions, while Mn, Co, Mo, Ni are less involved in oxidation-reduction reactions, and Zn is redox-inert. All of them are prone to form coordination complexes, such as zing fingers, heme, cobalamin (vitamin B12) and molybdenum cofactor (MoCo).

All mentioned trace elements, while maintained under homeostatic control, play vital roles in the human body. Most of the iron is utilized in the bone marrow for the synthesis of hemoglobin and red blood cells. Besides, iron is distributed to peripheral tissues through the bloodstream, where it is necessary for DNA synthesis, cell cycle progression, energy generation, heme synthesis, and the formation of iron-sulfur (Fe-S) clusters [28]. Zinc is necessary for the functioning of approximately 10% of the mammalian proteome, which contains zinc-binding domains, primarily zinc fingers [29]. These structures enable interactions with various biomolecules, including nucleic acids and other proteins, allowing zinc to regulate essential cellular processes such as DNA synthesis, gene regulation, enzymatic catalysis and apoptosis [30]. Moreover, zinc(II) ions play a signaling role, participating in phosphorylation and redox processes, and acts like an antioxidant by being a component of antioxidant proteins (for example, superoxide dismutase 1 (SOD1)) and metallothioneins [31]. Manganese is critically involved in energy metabolism by activating pyruvate carboxylase, isocitrate dehydrogenase, and glycosyl transferase, an enzyme required for mucopolysaccharide production. Also, there is a specific group of enzymes, vital specifically for the function of neurons and glial cells, while also playing roles in other tissues, which exhibits a strict dependence on Mn: superoxide dismutase 2, glutamine synthetase, arginase, pyruvate decarboxylase, and serine/threonine phosphatase [32]. Сopper is the critical cofactor of cytochrome-c-oxidase, influencing the mitochondrial electron transport chain, and is required for the activity of SOD1, same as Zn. Additionally, copper is essential for iron uptake and homeostasis, acting as a cofactor for ferroxidases like ceruloplasmin and hephaestin, which facilitate iron transport and prevent anemia [33]. Nickel is a component of essential enzymes like urease, hydrogenase, s-methyl coenzyme-M reductase, acetyl CoA synthase, CO dehydrogenase, Ni-superoxide dismutase, glyoxalase 1, and cis–trans isomerase. In parallel, molybdenum is central to the function of enzymes like xanthine oxidase and sulfite oxidase, crucial for purine metabolism and detoxification [34]. Cobalt is uniquely required as a core component of the cobalamin (vitamin B12), which is vital for DNA synthesis and neurological function.

Free ion concentration of these metals under normal conditions is low because their homeostasis is controlled by metal-binding and transporting proteins. The control begins when a metal enters the cell via specific influx systems: divalent metal transporter 1 (DMT1) for the majority of metals, the transferrin (TF)-transferrin receptor (TFR) complex for Fe and Mn, ZIP proteins (SLC39A) for Zn and Mn, calcium channels for Mn, Co and Ni [35], and copper transporter 1 (CTR1) for Cu. Molybdenum may decrease copper bioavailability by forming complexes with it or competing for uptake via transporters, while zinc and nickel may also act antagonistically [36,37]. After entry, in order to prevent an increase in free ion concentration, metals are bound by specific proteins, which are called metallothioneins (MTs). Moreover, glutathione is also able to bind various metals, while ferritin specifically sequesters iron, Atox1 and some other chaperones—copper. Excessive amounts of ions are exported by particular transporters: ferroportin (FPN) for Fe and Mn, ZnT transporters (SLC30A) for Zn, and ATPases ATP7A/B for Cu [32,37]. 

Metal Regulatory Transcription Factor 1 (MTF1) is primarily involved in controlling cadmium, zinc, and copper concentration in the cell. MTF1 serves as a transcription factor for metal-dependent proteins involved in cell and tissue development in accordance with the blood system. Ultimately, MTF1 is regulated by the cellular presence of the respective metal ions and stress conditions, including insufficient blood supply. It makes MTF1 one of the key genes responsible for tumor-associated angiogenesis and a target for further therapy approaches.

Dysregulation of metal levels often occurs in cancer, which frequently involves an increase in the labile pool of ions within the cell, resulting from impaired function of metal transporters or metal-binding antioxidant proteins. Free ion overload may lead to the excessive production of ROS, which further promotes carcinogenesis. This occurs primarily through the Fenton and Haber-Weiss reactions (•O_2_^−^ + H_2_O_2_ → •OH + OH^−^ + O_2_) in the presence of iron and copper ions. 

Cobalt, molybdenum, and nickel also facilitate ROS generation andinduce mutations in genes like KRAS, EGFR, and TP53, and by replacing magnesium ions in heterochromatin, causing its decondensation and subsequent chromosomal aberrations, respectively [35,36]. Manganese lacks Fenton activity and may competitively inhibit iron, but at the same time, its accumulation associates with poor prognosis in glioblastoma/melanoma and promotes migration via exosomal transport [38]. Similar to manganese, redox-inert zinc can inhibit Fenton chemistry by displacing redox-active ions from critical membrane sites, thereby preventing ROS formation [39]. Thus, zinc differs from the aforementioned metals, as its deficiency, rather than overload, is more commonly observed in cancer, especially esophageal [40]. Nevertheless, any dysregulation of zinc homeostasis can influence cancer development and progression by supporting cancer cell growth and survival, altering their sensitivity to apoptosis [39,40].

Enzymes regulating metal homeostasis and molecular targets of important signaling pathways are critically involved in cancer pathogenesis, with distinct roles in metastasis and multi-drug resistance (MDR). Pro-tumorigenic iron accumulation is driven by increased transferrin receptor 1 (TFR1) and hepcidin (which downregulates the exporter ferroportin (FPN1)), with alternative iron transport through increased lipocalin 2 (LCN2), which promotes metastasis and invasion, also inhibiting apoptosis [41]. Similarly, aberrant zinc homeostasis enhances cancer invasiveness and MDR. High metallothionein expression promotes MDR by sequestering chemotherapeutic drugs, neutralizing ROS, and donating zinc to transcription factors, while upregulated zinc transporters like ZIP7 hyperactivate growth pathways (such as EGFR, HER2) and ZIP10 enhances cell migration [42]. Stress-induced zinc release from zinc-finger motifs alters NF-κB, p53, and AP-1 activity to promote survival, and zinc finger transcription factors (for instance, Snail, ZEB) drive MDR by enhancing ABC transporter gene expression [43]. Manganese contributes to invasion by activating SOD2 in order to increase mitochondrial oxidative stress and by modulating epigenetic regulation through histone acetyltransferase (HAT) suppression and histone deacetylase (HDAC) activation [38]. Copper-dependent enzymes like MEK1, SOD1, COX, LOX participate in tumor growth and metastasis, and copper overload promotes MDR through reduced drug intake via lower CTR1 expression, impaired DNA repair via Atox1-induced expression of MDC1, and elevated drug efflux via ATP7A [44]. Cobalt overload supports cancer cells’ proliferation and angiogenesis via the stabilization and subsequent high activity of hypoxia-inducible factor 1 (HIF-1) [45].

### 2.3 Group 3: Non-Essential Trace Elements

Most metals of this group are often referred to as “heavy metals” due to their high atomic weight. Their presence inside the cells and the organism as a whole is minor, however, they still can have some specific function in biochemical pathways or be poisonous and toxic, which is a more common case.

Typically known poisonous and carcinogenic heavy metals include arsenic, cadmium, lead, mercury, and chromium (VI). Their carcinogenic mechanism involves prolonged oxidative stress caused by chronic ROS production via binding to thiol groups, displacement of essential metals, disruption of antioxidant defenses, DNA damage, and mitochondrial dysfunction [46,47]. Specifically, arsenic predominantly generates superoxide and hydrogen peroxide radicals, causing single- and double-strand DNA breaks and the formation of DNA adducts [46,48]. Cadmium mainly acts by binding to thiol groups of antioxidant enzymes and inducing lipid peroxidation, leading to oxidative stress without direct ROS generation [49]. The primary toxic effect of lead is the depletion of the cellular antioxidant pool, particularly glutathione (GSH) [50]. Mercury inhibits selenoenzymes, such as thioredoxin reductase, which are critical for maintaining antioxidant defenses [51]. Although the trivalent chromium form is essential, the hexavalent one is toxic to the cell. The key source of its toxicity is the reduction of Cr(VI) to Cr(III), resulting in hydrogen peroxide and other ROS generation [52].

Besides mentioned, there are inert metals, such as Au, Pt, Ir, and Ru, free ions of which are not typically prevalent in cells, but compounds with such elements exhibit toxic effects toward cancer cells.

**Table 1:** Metals in cellular homeostasis and cancer.

Group of Metals	Metal	Physiological Role	Role in Cancer (upon Dysregulation)
Group 1	Na	Creation of membrane potential	Apoptosis inhibition, cell cycle control, cell shape change
	K	Creation of membrane potential, glycolysis, protein synthesis, cell cycle progression	Immune response alteration
	Mg	Cofactor for enzymes, “a shield” for negative charges on proteins	Stimulation or inhibition of tumor growth, depending on conditions
	Ca	Secondary messenger in signalling pathways, a trigger of cellular responses	Tumor survival and growth support
Group 2	Fe	Heme production, formation of Fe-S clusters, DNA synthesis, cell cycle progression, energy generation	Excessive ROS production through Fenton and Haber-Weiss reactions, metastasis and invasion promotion
	Zn	Structural and catalytic cofactor, signalling role	Tumor survival and growth support, MDR development
	Cu	Catalytic cofactor, iron uptake and homeostasis support	Excessive ROS production through Fenton and Haber-Weiss reactions, MDR development
	Mn	Energy metabolism support, catalytic cofactor	Promotion of migration via exosomal transport, mitochondrial oxidative stress increases
	Co	Component of the cobalamin	ROS-induced mutations in gene occurrence, replacement of magnesium ions in heterochromatin, and angiogenesis support
	Ni	Catalytic co-factor	ROS-induced mutations in gene occurrence, replacement of magnesium ions in heterochromatin
	Mo	Catalytic co-factor	No established role in cancer
Group 3	As, Cd, Pb, Hg, Cr (VI)	No beneficial biological function, toxic	Сhronic ROS production, displacement of essential metals, disruption of antioxidant defenses, DNA damage, mitochondrial dysfunction
	Au, Pt, Ir, Ru	Biologically inert	Core of chemotherapeutic agents

Note: MDR, multidrug resistance; ROS, reactive oxygen species.

## 3 Metal-Based Therapeutics and Their Mechanisms of Action

Metals have been used for drug purposes since ancient times [53]. Their versatile properties can be utilized for the development of a great variety of chemical compounds. Despite this versatility, only a minor fraction of chemotherapeutic drugs are present now on the market compared to the drugs of other chemical origins [4]. Furthermore, even this number is often downsized to several key drugs used in clinics, among which cisplatin is the most famous. This often leads to the misconception about metal-based drugs, which manifests itself in the generalization of cisplatin properties to all therapeutics of this category. In this section, we will explore the mechanisms of action for existing metal-based drugs and a variety of their properties and forms. The previous classification of metals from Section 2 will be used to enable easy comparison between the physiological role of the metal and its translation into anticancer treatment.

### 3.1 Group 1: Essential Biogenic Metals (Na, Mg, Ca, K)

The high activity and importance of alkali and alkaline earth metals for all living cells, as well as the prevalence and similarity of the metabolism of these metals in normal and tumor cells, make it difficult to develop stable drugs based on sodium, potassium, calcium or magnesium [54]. However, these elements have found their application in cancer therapy as modulators of antitumor response or supportive treatment that helps alleviate the symptoms or adverse effects of the main course of chemotherapy, e.g., dyselectrolytemias [55].

While most drug molecules exist as sodium or potassium salts, the ions in this case do not possess any therapeutic activity but rather serve as a counterion, hedging critical functional groups. Due to its activity, sodium is not properly accountable for creating stable coordinating complexes or nanoparticles. Represented sodium drugs, including mostly metabolite counterparts, like methotrexate sodium or talaporfin sodium [56], and specific nanostructures, like sodium selenite [57]. 

Potassium acts similarly to sodium, enhancing drug solubility as a counterion in compounds like potassium oxonate or potassium quercetin-5′-sulfonate. The element has also found its application in the stimulation of the immune system and combined modalities with immunotherapy [58].

That cannot be said about calcium-based compounds, whose major advantage is their full physiological biocompatibility, stability, and biodegradability. One of the crucial directions in calcium-based therapeutics is the development of pH-responsive nanomaterials for targeted delivery [59]. Calcium phosphates, calcium carbonates, calcium silicate, and calcium fluoride are widely utilized for these purposes. Various forms of CaX NPs are now under research or development [59,60]. These materials can be applied for the treatment of various types of cancer, including breast and prostate types [61]. Major concerns regarding calcium-based therapeutics and treatment modalities are systemic toxicity (for nanoparticles), variability in calcium channels expression profile, and rapid clearance due to high biocompatibility [62]. 

In many aspects, magnesium shares the properties of calcium. Its usage is also concentrated on the magnetic alloys, pH-responsive nanomaterials and nanoparticles [63]. The advantages of Mg-based therapeutics include their high biocompatibility and biodegradability, while concerns mainly concentrate around the high reactivity of the metal, its dual role in cancer (see above), and limited data from clinical trials [64]. For manganese, there are also several nanosystems that are currently under development for the treatment of cancer of various origins, particularly breast and lung cancer [65]. Some works also describe current efforts to develop Mg-based nanomaterials for the delivery of anticancer drugs and their targeted release [66].

It is worth noticing that ion channels for all elements of this group are actively studied as a suitable target for cancer treatment. These compounds are not metal-based per se, but they possess the activity allowing them to alter the intracellular transport of critical ions. There are numerous pharmacological substances targeting sodium [67], potassium [68], or calcium [69] ion channels. The opposite group of compounds are often called ionophores since they are able to bind metal ions allowing their alleviated transport across the plasma membrane. This process increases the concentration of metal ions inside the cell that results in overload and disruption of critical processes [70]. These two classes of drugs while beyond the scope of this review attract ultimate research interest in recent years.

### 3.2 Group 2: Essential Trace Elements (Fe, Zn, Cu, Mn, Co, Ni, Mo)

#### 3.2.1 Ferrum

Two main strategies, regarding iron presence in the tumor and body, are the induction of iron-specific cell death (ferroptosis) and the depletion of tumor-essential iron (Fig. 2). Under intact human metabolism, iron is used as the enzyme cofactor performing key cellular functions: replication, carbohydrate metabolism, electron chain transfer, inactivation of toxins and ROS. Prevention of essential iron entering the tumor is a distinct strategy that increases tumor vulnerability or indirectly inhibits its growth.

Iron chelator, Deferasirox, used primarily for the treatment of iron overload conditions, is sold under the brand of Exjade [71]. It showed activity against solid tumor models—lung tumor xenografts—in pre-clinical studies. Its antitumor activity stimulates senescence and pro-apoptotic outcomes in leukemia cells: upregulation of p21 and N-myc, downregulation of cyclin D1 levels [72].

Deferoxamine or desferrioxamine was used primarily as a predecessor of Deferasirox for the treatment of diseases related to iron overload [73]. Exploratory research revealed the contributive role of iron depletion in ovarian tumor cells by Deferoxamine with combinational chemotherapy.

Deferiprone, drug approved for treating iron overload during diseases and to dampen conditions at blood transfusion [74]. Current studies aim to investigate its possibilities for the treatment of neurological diseases and malignancies [74].

Fe-based nanoparticles for tumor treatment are mostly represented by magnetically or optically activated agents for magnetothermal or photothermal therapy (Fig. 2). The magnetic properties of iron oxide is a key property allowing its use for therapeutic and diagnostic purposes [75]. The general mechanism of such compounds is as follows: molecules or particles accumulate inside a solid tumor; near-infrared light excites iron oxide; iron oxide converts light energy into heat; and the local tumor experiences hyperthermia, which causes its elimination. Ferumoxytol is an approved drug primarily serving for the treatment of iron deficiency. It consists of a superparamagnetic iron oxide core surrounded by carboxymethyl dextran and exhibits immune-modulating function by altering the polarization of tumor-associated macrophages. Iron oxide nanoparticles found to be capable of reprogramming tumor-associated macrophages into a pro-inflammatory state [76].

#### 3.2.2 Zinc

Zinc-related approaches in cancer therapy consider both exploiting this metal as a drug component and controlling its concentration in the tumor or the whole body.

Zinc serves as a cofactor for the regulation of angiogenesis-related genes, thus zinc-depriving strategies are investigated to inhibit the growth of solid tumors [77,78]. A class of metal-chelating compounds is quinols. Studies of clioquinol showed its potency in inhibiting the proliferation of prostate cancer and myeloid leukemia [79]. Zinc oxide nanoparticles induce ROS upon the directed optical or ultrasound stimulation, which is exploited for the treatment of solid tumors [80]. An intriguing application of zinc-titanium oxide nanoparticles is a delivery container for an antigen in a dendritic-cell-based anticancer vaccine [81]. As a delivery agent, zinc oxide NPs can also be tracked with MRI techniques and serve as a contrast agent [82]. Zn-complexes with nitrogen donor ligands are used as agents in a photo-dynamic therapy. These zinc-containing compounds usually possess stronger cytotoxicity in comparison with conventional chemotherapeutic compounds [83]. DNA-binding properties of zinc are being used in combination with pyridine complexes. These molecules exhibited sufficient effect in DNA intercalation [84].

#### 3.2.3 Copper

Regarding the aforementioned copper role in normal and cancer metabolism, following strategies for tumor treatment are used: copper depletion [85], excessive copper influx [86], and induction of copper-specific cell death [87,88].

The combination of copper chelators was used as a therapeutic approach to increase the potency of combinational chemotherapy [89]. Elesclomol increases intracellular Cu^2+^ concentration, leading to ROS induction, mitochondria and DLAT complex impairment related to cuproptosis events [90]. The ultimate cellular outcome of cuproptosis is not sufficiently described through well-established cell death hallmarks and morphological features [91]. Authors make a careful suggestion that the ultimate cellular outcome of cuproptosis is cell death due to energy depletion, possibly a necrotic type.

As a transition metal, copper occupies a place in the field of NPs synthesis for therapeutic and diagnostic purposes [92]. Exogenic copper reduction from Cu^2+^ to Cu^1+^ disrupts intracellular systems, maintaining the reduction-oxidation balance. It is common for tumors to change their RedOx balance due to mutilation of the intact cell metabolism. Distinctive changes in the antioxidant system are actively exploited to treat solid tumors. Copper-containing NPs and lightweight organic complexes are used to mediate ROS-dependent cytotoxicity in tumors [93,94]. The mechanism of ROS-mediated tumor toxicity of copper, besides cuproptosis, is based on the distortion of glutathione (GSH) or GPX4 antioxidant system [95].

#### 3.2.4 Manganese

Manganese (Mn) has emerged as a highly versatile element in the design of advanced anticancer formulations, which are primarily grouped around manganese-based nanoparticles [96]. These include sophisticated designs like Mn-coordinated NPs, where manganese ions are chelated by organic ligands; Mn-doped Prussian blue NPs, which leverage a classic framework for enhanced functionality; and mesoporous Mn nanocarriers (NCs), prized for their high drug-loading capacity. A significant advantage of these Mn-formulations is their role as potent theranostic agents, integrating diagnostic capabilities with therapeutic action via photothermal activity [97]. Beyond direct killing, manganese is a powerful immune modulator able to activate the cGAS-STING pathway [98]. This activation occurs as Mn^2^^+^ ions are released in the tumor microenvironment, potentially triggering a robust antitumor immune response and provoking immunogenic cell death. The efficacy of these platforms hinges on their ability to generate cytotoxic ROS and localized heat, which collectively contribute to tumor microenvironment reprogramming, shifting it from immunosuppressive to immunogenic, and ultimately enhancing the overall antitumor outcome [99]. Manganese-based NPs are used in combinational therapy with convenient chemotherapeutics [100]. The multi-faceted role of the manganese ion in the immune pathways of a cell is exploited in constructing co-loaded particles for reprogramming of the tumor microenvironment [101].

#### 3.2.5 Cobalt

Cobalt (Co) offers distinct mechanisms for anticancer therapy, primarily through its radioactive and chemical properties. Historically, the radioisotope ^60^Co irradiation therapy has been a cornerstone of external beam radiotherapy. More recently, cobalt redox activity has been harnessed for chemodynamic therapy, where cobalt-based nanoparticles (e.g., CoO NPs) can catalyze the Fenton-like reaction within the tumor microenvironment to generate cytotoxic reactive oxygen species. A prominent strategy for enhancing selectivity involves combinational therapies, particularly the use of cobalt(III)-cyclam prodrugs. These inert complexes are designed to be activated by reduction in the hypoxic tumor milieu, releasing cytotoxic ligands; this activation can be synergistically enhanced by co-administration of ascorbate, which serves as a reducing agent [102]. The efficacy of these formulations is deeply tied to microenvironment modulation, as the dysregulated biochemistry of tumors (e.g., hypoxia, elevated glutathione) provides the ideal conditions for activating cobalt(III) prodrugs and for the catalytic activity of CoO NPs, offering a targeted approach to cancer treatment.

#### 3.2.6 Nickel

Biomolecular research data suggest a distinct role of nickel and its compounds in carcinogenesis [103,104]. Despite that fact, careful experiments reveal the role of nickel as a coordinating atom for small organic complexes exhibiting anticancer potential, including nickel(II) complexes of thiosemicarbazones and compounds like nickel(II)-N-(2-hydroxyacetophenone)-glycinate (NiNG), which have demonstrated promising efficacy [105]. 

More advanced nanostructures, such as nickel-based single-atom-metal-clusters, are being engineered for enhanced specificity and efficacy [106]. These formulations function as potent tumor growth inhibitors through multifaceted mechanisms [107]. A primary mode of action is the induction of oxidative stress, which contributes to extensive DNA damage and subsequent DNA synthesis inhibition, halting cellular proliferation. Beyond genomic targeting, these agents engage in cellular pathways targeting, with some exhibiting potent activity as proteasomal deubiquitinase inhibitors. This disruption of protein degradation pathways leads to the accumulation of misfolded proteins and ultimately triggers apoptotic cell death, highlighting the potential of nickel for targeting non-genomic vulnerabilities in cancer cells. The promising therapeutic role of nickel-based compounds is limited since nickel itself is a carcinogenic factor [108].

#### 3.2.7 Molybdenum

Molybdenum (Mo) garnered significant interest in nanomedicine for its potent and versatile applications in cancer therapy, particularly in photothermal therapy. 

Key materials such as MoS2, which strongly absorb light in the near-infrared (NIR) spectrum, and MoO_2_ have been extensively studied for their efficient light-to-heat conversion [109]. The functionality of these materials is further enhanced through surface engineering, leading to the development of aptamer-modified nanosheets that improve tumor targeting and cellular uptake. These Mo-based nanosheets serve as excellent platforms for combination with photothermal molybdenum-containing drugs, integrating hyperthermia with other treatment modalities [110]. Beyond their photothermal capabilities, molybdenum is also utilized in the construction of nanocarriers for chemotherapeutics. These nanocarriers can deliver traditional drugs directly to the tumor site, and the release can be triggered by the acidic tumor microenvironment or the localized heat generated during photothermal therapy, enabling a powerful synergistic attack on cancer cells [111].

### 3.3 Group 3: Non-Essential Trace Elements

Elements from group 3 historically were the most used in cancer treatment, with cisplatin (and platinum-based drugs) being the best known and widely applicable substance suitable for a variety of clinical cases. The major obstacle in utilizing elements of this group and drugs based on their basis is the lack of biocompatibility and biodegradability, which often results in side effects that can last for years [112,113]. Heavy elements cannot be incorporated in natural biochemical circuits, which leads to their accumulation in various sites, usually bones, kidneys, liver, and brain. Nevertheless, unique properties of the elements of this group make them indispensable for the development of anticancer drugs even beyond classic cytotoxic substances. New therapeutic modalities include formulations for theranostics allowing real-time controlled treatment of malignancies. A lot of elements of this group have found their application in radiotherapy, because heavy elements usually have several unstable isotopes that are a perfect match for the treatment or diagnostic purposes [114]. Below, we will emphasize the progress made in the development of therapeutic formulations based on specific elements that currently have (or might have in the future) the biggest potential in the field.

#### 3.3.1 Platinum

Platinum-based drugs are the best-known metal-based anticancer agents. Cisplatin was discovered in the second half of the 20th century, and ever since, 5 more formulations appeared on the market, with several more going through clinical trials [115]. As was mentioned before, the major problem with platinum-based drugs is their poor bioavailability, which results in side effects. Another problem is the formation of drug resistance to cisplatin, which is reported for ovarian cancer and other types. To address this issue, new compounds have been developed, e.g., picoplatin and satraplatin, which are currently in clinical trials [116,117]. Mechanism of their action is similar, DNA-damage via crosslinking and subsequent apoptosis, however the type of damage that they inflict is harder to repair than that of cisplatin.

#### 3.3.2 Ruthenium

Ruthenium-based drugs were under development almost at the same time as cisplatin but the success of the latter outshone these substances [118]. Ruthenium-based compounds demonstrated several key advantages compared to widely used cis-platinum analogs. One of the major advantages is enhanced accumulation of active Ru(II)-complexes inside tumor cells due to a reduced environment of tumor tissue, caused by hypoxia, low pH, or elevated glutathione levels. Ruthenium complexes (e.g., NAMI-A, KP1019) are notable for their reduced toxicity and unique features, including ROS generation and modulation of the tumor microenvironment with several candidates in advanced clinical trials [119].

#### 3.3.3 Iridium

Iridium is a highly promising element for developing anticancer therapeutics since it shows versatile chemical properties, allowing the creation of unique compounds. This metal possesses phosphorescent properties and can exist in various oxidation states (from −3 to +9) with +3 being the most stable under physiological conditions. Moreover, iridium itself is chemically inert toward biological systems, which is why it causes no side effects, but makes the process of drug development harder [120]. Some findings indicate that novel iridium(III) complexes can be designed to trigger apoptosis in cancer cells [121], while iridium guanidine complexes demonstrate drastic activity against cisplatin-resistant ovarian cancer cells [122]. Furthermore, iridium-based compounds often cause mitochondrial dysfunction, inhibition of protein kinases (MAPK, PI3K), and ROS generation [121], which makes them alternatively attractive for novel drug development.

#### 3.3.4 Aurum

Gold has attracted significant research attention from the field of drug delivery systems. While inert, the element can serve as a vehicle for other substances, bringing them to the site needed [123]. Recent studies indicate numerous advantages of nanogold over other nanomaterials, particularly, highly optimized protocols for the production of gold nanoparticles of various sizes and shapes, featuring unique properties, and the possibility to modify the surface of the nanoparticles with different functional compounds, boosting targeting and therapeutic properties [124]. It was also found that the cytotoxicity of gold nanostructures depends on their shape. While gold nanospheres are virtually inert, gold nanorods or gold nanostars can be severely toxic for cells, including cancer [125]. Moreover, various shapes allow gold NPs to possess distinct photoactivity, which makes them suitable for PDT [126]. These properties make it possible to use gold-based nanoplatforms for a variety of different tasks.

#### 3.3.5 Plumbum and Vanadium

Other important representatives of the third group of elements include lead and vanadium, both of which have a distinct influence on a human organism. Lead is well-known for its toxic nature toward living cells [127]. The metal realizes this ominous potential through inhibition of critical enzymes related to DNA repair, ROS generation, and direct DNA and chromosome damage [128]. However, lead-based compounds are in development with some nanoformulations appearing in the field, e.g., lead-based coordination polymer with polyvinyl alcohol (PVA) and 3-carboxypropyltriethoxysilane (CPTES) [129]. This composite system allowed controlled drug release, significantly decreasing HCC cell survival by downregulating ENSA expression.

The role of vanadium in the body is complicated and understudied. The geometry of this metal in tetrahedral formation (vanadate (V)) resembles that of the phosphate ion, which allows the former to substitute for it in some reactions [130]. This phenomenon is particularly important in the context of kinase functioning, which is why vanadium may play a role in diabetes and cell signaling [131]. This is also the reason why vanadium is used for cancer treatment in the form of organic complexes or nanoformulations. These agents can influence the activity of tyrosine phosphatases and phosphorylases, which leads to the induction of apoptosis. DNA cleavage and oxidative stress were also reported [132]. Currently, the lack of knowledge on vanadium biology and its role in a human organism limits the development of new, more targeted and sophisticated compounds.

### 3.4 Metal-Organic Framework (MOFs)

Beyond discrete complexes and nanoparticles, metal-organic frameworks (MOFs) represent a frontier in inorganic-organic hybrid materials, functioning as reactive and functional polymers with precisely engineered porosity and composition [133]. Their crystalline structure, built from metal-ion nodes (e.g., Fe, Zn, Cu, Zr, Mn) and organic linkers, creates a versatile platform for cancer theranostics. A key advantage is their high surface area and tunable chemistry, which enables exceptional drug loading and stimuli-responsive release within the tumor microenvironment (TME). For instance, zeolitic imidazolate framework-8 (ZIF-8), a Zn-based MOF, is stable at physiological pH but rapidly degrades in acidic tumor microenvironment, releasing encapsulated chemotherapeutics with high specificity [134,135]. Similarly, Fe-based MOFs (e.g., MIL-100) and Cu-based MOFs (e.g., HKUST-1) not only deliver drugs but also act as intrinsic nanozymes, catalyzing the conversion of tumor H_2_O_2_ into cytotoxic hydroxyl radicals via sustained Fenton reactions for potent chemodynamic therapy (CDT) [136]. More advanced theranostic designs incorporate imaging agents; Zr-based MOFs (e.g., UiO-66) loaded with gadolinium or radioisotopes can provide contrast for MRI or act as radiosensitizers while delivering therapeutic payloads [137]. Despite their promise, challenges such as controlled biodegradation, long-term *in vivo* stability, and scalable synthesis remain active areas of investigation [133].

## 4 Modalities of Metal-Based Therapy

Metal-based therapies represent a powerful and expanding frontier in oncology, offering a compelling alternative to conventional non-metal-based drugs. It is important to understand the variety of their actions in order to expand the area of potential applications. The utility of metal-based compounds stems from the unique physicochemical and redox properties of metal ions, which enable diverse mechanisms of action, often capable of bypassing common resistance pathways such as drug efflux pumps and enhanced DNA repair [138,139]. The following section details the application of metal-based agents across various therapeutic modalities, highlighting their distinct mechanisms (see Table 2) and clinical examples, and comparing them to more classical approaches.

**Table 2:** Principles/mechanisms of action for the main therapeutic modalities.

Modality	Metal-Based	Non-Metal-Based
Conventional therapy	Direct DNA damage: formation of irreversible, cytotoxic DNA adducts and cross-links (e.g., Pt-based drugs)	DNA intercalation: insertion of planar molecules between DNA base pairs, disrupting replication
	Redox dysregulation: catalysis of Fenton-like reactions (Fe, Cu) to generate ROS, inducing oxidative damage to lipids, proteins, and DNA (cuproptosis, ferroptosis)	Mitotic arrest: stabilization or destabilization of microtubules to halt cell division (e.g., taxanes, vinca alkaloids)
	Enzyme inhibition: targeting specific enzyme active sites (e.g., thioredoxin reductase by Au(I) complexes), vanadium	Topoisomerase inhibition: trapping of topoisomerase complexes, leading to DNA strand breaks (e.g., anthracyclines, etoposide)
	Multi-targeted action: simultaneous induction of apoptosis, ferroptosis, and immunogenic cell death (e.g., cisplatin, Fe-based NPs, elesclomol)	Metabolic disruption: inhibition of key metabolic enzymes (e.g., antimetabolites like 5-FU, methotrexate)
Radiotherapy	Internal radionuclide therapy: delivery of high-energy α/β-particle-emitting isotopes (e.g., ^223^Ra, ^177^Lu) directly to tumors, causing localized, irreparable DNA double-strand breaks	External beam radiation: use of photons (X-rays) or particles (protons, carbon ions) to generate DNA-damaging free radicals (ROS) systemically within the tumor volume. Stereotactic radiosurgery (SRS)—gamma knife, cyber knife etc.
	Radiosensitization: use of high-Z elements (e.g., Au, Pt NPs) to enhance local energy deposition and ROS generation from external beam radiation	
Phototherapy	Photothermal therapy (PTT): conversion of near-infrared light to localized heat using plasmonic metal nanoparticles (e.g., Au, CuS), causing hyperthermic ablation of tumor tissue	Organic photothermal therapy: use of organic dyes (e.g., indocyanine green (ICG)) or carbon-based materials (e.g., CNTs) to convert light to heat, albeit often with lower conversion efficiency than metals
	Photodynamic therapy (PDT): light-triggered generation of cytotoxic singlet oxygen and ROS from photosensitizing metal complexes (e.g., Ru, Ir)	
Immunotherapy	Innate immune activation: acting as pathogen-mimicking agents or STING pathway agonists (e.g., Mn^2+^) to reverse immunosuppression and promote dendritic cell/T-cell activation	Checkpoint blockade: use of monoclonal antibodies to inhibit T-cell suppressor receptors (e.g., anti-PD-1, anti-CTLA-4)
	Macrophage reprogramming: repolarization of tumor-associated macrophages (TAMs) from pro-tumor M2 to anti-tumor M1 phenotype (e.g., with Fe, Cu-based NPs)	Engineered cell therapy: genetic modification of T-cells to express chimeric antigen receptors (CAR-T) for targeted tumor cell killing
	Immunogenic cell death (ICD): inducing calreticulin exposure, ATP release, and HMGB1 secretion to stimulate an adaptive immune response (Fe, Cu)	
Targeted therapy	Protein-targeted inhibition: design of complexes to inhibit specific oncogenic proteins or pathways (e.g., kinase inhibition by Ru/Au complexes, p97 inhibition by CuET)	Small molecule inhibitors: competitive inhibition of oncogenic kinase active sites (e.g., EGFR, BCR-ABL inhibitors).
	Metallochaperone therapy: reactivation of mutant tumor suppressors (e.g., Zn-based reactivation of mutant p53)	Monoclonal antibodies: blockade of growth factor receptors or ligands (e.g., trastuzumab anti-HER2, bevacizumab anti-VEGF).
	Exploitation of metal metabolism: selective targeting of cancer cells with dysregulated metal homeostasis (e.g., cuproptosis induction in Cu-high cancers)	

Note: 5-FU, 5-fluorouracil; CTLA, cytotoxic T-lymphocyte associated protein; CuET, copper(II) diethyldithiocarbamate; NPs, nanoparticles; PD-1, programmed cell death 1; VEGF, vascular endothelial growth factor.

Traditional metal-based chemotherapy, as was mentioned earlier, pioneered by platinum drugs, primarily functions through direct DNA damage. Cisplatin, carboplatin, and oxaliplatin form covalent bonds, particularly at the N7 position of guanine, leading to intra- and interstrand DNA crosslinks that block replication and trigger apoptosis [140]. Beyond platinum, other metals employ novel strategies. Ruthenium(III) complexes like NAMI-A and KP1019 are under clinical investigation for their distinct profiles [119]. Vanadium-based species act as potent inhibitors of phosphatases and kinases by mimicking substrates [141]. Iron is leveraged in ferrocifen derivatives, which generate lipid reactive oxygen species (ROS) to induce ferroptosis, a form of regulated cell death [142]. Similarly, copper-based Casiopeinas catalyze hydroxyl radical formation via Fenton-like reactions, impairing the Nrf2/KEAP1 antioxidant pathway and promoting apoptosis [143]. Other copper complexes, such as Cu(II) phenanthroline-phenazine, show promise in overcoming cisplatin resistance [144]. Additionally, zinc oxide nanoparticles (ZnO NPs) induce mitochondrial damage, apoptosis, and ferroptosis [145]. 

Metal-organic frameworks (MOFs) exemplify the convergence of conventional and novel modalities within a single nanostructure. For example, a Mn-based porphyrinic MOF can simultaneously function as a carrier for doxorubicin, a catalyst for O_2_ generation to relieve hypoxia, and a photosensitizer for photodynamic therapy, attacking tumors through combined chemotherapy, oxygenation, and ROS generation [137]. This multifunctionality underscores the capacity of MOFs to integrate diagnosis and multiple treatment mechanisms, moving beyond single-mechanism drugs.

In contrast, non-metal chemotherapeutics operate through organic mechanisms, including microtubule stabilization (e.g., taxanes), DNA intercalation and topoisomerase inhibition (e.g., anthracyclines), and antimetabolite action (e.g., 5-fluorouracil) [146].

In radiotherapy, metal-based approaches fundamentally differ from conventional external beam radiation by delivering radiation-emitting isotopes directly to the tumor microenvironment. These strategies often employ metal radionuclides chelated to targeting molecules, such as peptides or antibodies, which allows for precise localization. Once accumulated, these isotopes emit high-linear energy transfer (LET) particles, such as alpha (α) or beta (β) particles, which cause highly concentrated and lethal DNA damage, primarily in the form of double-strand breaks [147]. A prominent example of an α-emitter is Radium-223 (^223^Ra) dichloride (Xofigo^®^), which naturally targets bone metastases and emits α-particles to eradicate cancer cells with high efficacy and limited range, sparing surrounding healthy tissue [148]. In the realm of β-emitters, Lutetium-177 (^177^Lu) has gained significant clinical traction, particularly in the form of ^177^Lu-PSMA (Pluvicto^®^) for treating metastatic prostate cancer, where it delivers targeted radiation upon binding to the prostate-specific membrane antigen [149]. Beyond direct radiation emission, metals also serve ancillary roles; for instance, the iron oxide nanoparticle ferumoxytol can be utilized for MRI-guided hyperthermia to synergistically enhance the effects of traditional radiotherapy [150].

In contrast, non-metal-based radiotherapy primarily relies on external sources of high-energy ionizing radiation, such as photon or electron beams from linear accelerators (LINACs) [151] or particle therapy with protons and carbon ions [152]. A classic non-metal radiopharmaceutical is Iodine-131 (^131^I) MIBG, used for the treatment of thyroid cancer by leveraging the thyroid gland’s natural iodine uptake [153].

Phototherapy (PTT/PDT) capitalizes on the unique optical properties of certain materials to ablate tumor tissue. Metal-based agents, particularly noble metal nanoparticles, are exceptionally effective in this modality due to their strong localized surface plasmon resonance, which enables them to efficiently absorb near-infrared (NIR) light and convert it into heat. Gold nanostructures, such as nanoshells and nanorods, have been extensively studied and have demonstrated the ability to achieve complete tumor ablation in preclinical mouse models due to their tunable NIR absorption [154,155,156]. Other metallic nanoparticles, including triangular silver NPs (AgNPs), palladium NPs and copper sulfide NPs (CuS), are also in advanced preclinical development for combined PTT and radiosensitization, showcasing the latitude of metals in light-mediated therapies [157,158,159]. 

On the other hand, non-metal photothermal agents are typically carbon-based or organic. These include carbon nanotubes (CNTs), which strongly absorb NIR light, conductive polymers like polydopamine, and organic dyes such as indocyanine green (ICG), which is already used in clinical applications. While these non-metal compounds are effective, metal-based nanoparticles often offer superior photothermal conversion efficiencies and greater potential for functionalization into other drugs.

The immunomodulatory potential of metals is a rapidly emerging frontier in cancer treatment, offering strategies to stimulate the immune system against tumors. Metals can act as potent innate immune activators or modifiers of the tumor microenvironment. For example, manganese (Mn^2^^+^), in the form of MnCl_2_, has been shown to potentiate the cGAS-STING pathway, a key cytosolic DNA-sensing route that triggers a robust type I interferon response and enhances antitumor immunity [160]. Furthermore, certain metal-based nanoparticles can directly reprogram immune cells; iron oxide nanoparticles like ferumoxytol have demonstrated the ability to repolarize immunosuppressive M2 macrophages into the pro-inflammatory, antitumor M1 phenotype in preclinical models [161]. Similarly, Ruthenium(II) and Rhodium(II) complexes are being investigated as experimental agents that can induce immunogenic cell death in tumor cells and modulate the function of tumor-associated macrophages (TAMs) [162]. More advanced formulations, such as HSA-C4 NPs and elesclomol-copper NPs, continue to build on this strategy by effectively converting M2 macrophages to M1 states, thereby overcoming the resistant tumor microenvironment [163,164]. This metal-based approach to innate immune activation contrasts with the dominant non-metal immunotherapies, which primarily focus on enhancing adaptive immunity [165].

“Conventional” methods of immunotherapy include checkpoint inhibitor monoclonal antibodies that block inhibitory receptors like PD-1 (e.g., nivolumab) or CTLA-4 (e.g., ipilimumab) on T-cells, and engineered cellular therapies such as CD19-targeting CAR-T cells (e.g., tisagenlecleucel), etc. [166,167].

Finally, the principle of targeted therapy—hitting specific molecular drivers of cancer—is now being powerfully applied through metal-based compounds, which can be engineered for high specificity. These agents are designed to interfere with oncogenic proteins or pathways in a precise manner. A compelling example is the use of manganese (Mn), which has been shown to selectively degrade the Golgi protein GOLIM4 in cancers with 3q amplification, thereby blocking a critical pro-tumor secretory pathway [168,169]. Another sophisticated strategy involves the repurposing of the old drug disulfiram; it acts as a prodrug that chelates copper in the body to form the active complex CuET. This complex specifically inhibits the p97-UFD1-NPL4 complex, a key component of the protein degradation machinery, leading to proteotoxic stress that is particularly lethal to cancer cells [170]. In the realm of precision oncology, zinc metallochaperones represent a highly targeted approach, designed to selectively reactivate specific, common mutant forms of the p53 tumor suppressor protein (e.g., p53^R175H^), thereby restoring a critical cellular defense mechanism [171]. Additionally, other metal complexes, such as those based on ruthenium or gold, are in investigational stages and are being engineered to bind and inhibit specific kinases or cell surface receptors [172].

The expanding arsenal of metal-based targeted therapies complements the well-established non-metal targeted drugs, which include small-molecule tyrosine kinase inhibitors (e.g., osimertinib for EGFR-mutant lung cancer, imatinib for CML) and monoclonal antibodies (e.g., trastuzumab targeting HER2) [173,174,175]. Table 3 summarizes examples of the most significant representatives of each type of compound by therapeutic modality.

**Table 3:** The main groups of compounds for each type of therapeutic modality.

Modality	Metal-Based	Non-Metal-Based
Conventional therapy	Platinum salts: cisplatin, carboplatin, oxaliplatin Ru(III) complexes: NAMI-A, KP1019 Vanadium: oxidovanadium(V) Iron-containing: ferrocifen derivatives Copper: casiopeinas, Cu(II) phenanthroline-phenazine complexes Zinc oxide NPs: ZnO	Taxanes: paclitaxel, docetaxel Anthracyclines: doxorubicin, mitoxantrone Antimetabolites: 5-fluorouracil (5-FU), methotrexate Vinca alkaloids: vinblastine, vincristine, vindesine Topoisomerase inhibitors: etoposide, topotecan
Radiotherapy	α-emitter: radium-223 (Xofigo^®^) β-emitter: lutetium-177-labeled agents (e.g., 177Lu-PSMA, Pluvicto^®^) MRI-guided hyperthermia agent: ferumoxytol (iron oxide nanoparticle)	Photon/electron therapy (LINAC X-rays) Particle therapy: protons, carbon ions I-131 MIBG therapy (for thyroid cancer)
Phototherapy	Gold NPs: nanoshells, nanorods Silver NPs: triangular for PTT/radiosensitization Palladium NPs: cubes, octahedron Copper NPs: CuO, CuS	Carbon-based: carbon nanotubes (CNTs) Organic polymers: polydopamine, conductive polymers Organic dyes: indocyanine green (ICG) Phosphorus: black phosphorus nanosheets
Immunotherapy	Iron oxide NP: ferumoxytol Manganese salts: MnCl_2_ Ru/Rh complexes: dirhodium paddlewheel complexes and ruthenium complexes Copper: elesclomol-copper NPs, HSA-C4 NPs	Checkpoint inhibitors: nivolumab (anti-PD-1), ipilimumab (anti-CTLA-4) CAR-T therapy: tisagenlecleucel (anti-CD19)
Targeted therapy	Manganese: MnCl_2_ Ru or Au: investigational kinase/receptor binder complexes Copper: CuET (disulfiram) Zinc: ZMCs (metallochaperones)	Tyrosine-kinase inhibitors (TKIs): osimertinib (EGFR), imatinib (BCR-ABL), Vemurafenib (BRAF) Monoclonal antibodies: trastuzumab (anti-HER2), bevacizumab (anti-VEGF), rituximab (anti-CD20)

Note: 5-FU, 5-fluorouracil; BRAF, serine/threonine-protein kinase B-Raf; CTLA-4, cytotoxic T-lymphocyte associated protein 4; HSA, human serum albumin; LINAC, linear particle accelerator; MIBG, metaiodobenzylguanidine; MRI, magnetic resonance imaging; NAMI-A, ruthenium-based imidazolium salt; NPs, nanoparticles; PD-1, programmed cell death 1; PSMA, prostate-specific membrane antigen.

Metal-based agents leverage unique physicochemical and redox properties to enable multimodal mechanisms of action, ranging from direct DNA damage and redox stress to immune modulation and targeted pathway inhibition. Their capacity to integrate into diverse therapeutic modalities—radiotherapy, phototherapy, immunotherapy, and targeted therapy—provides a versatile platform for overcoming conventional resistance mechanisms and enhancing the precision and efficacy of cancer treatment. 

## 5 Overcoming Multidrug Resistance via Metal-Based Drugs

**Figure 3:**
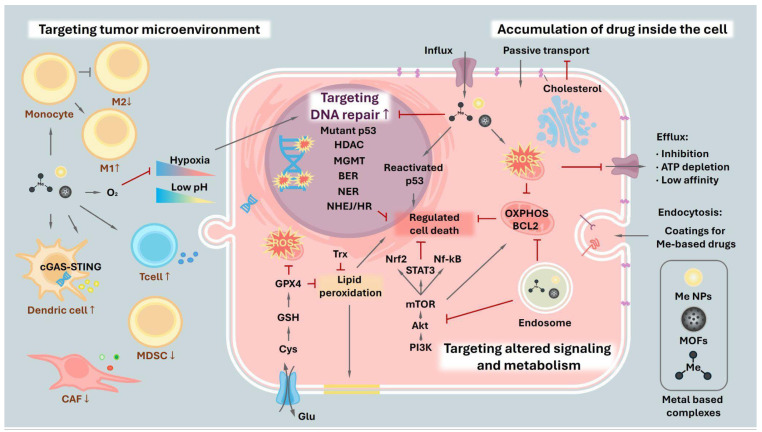
Metal-based compounds counter major mechanisms of therapy resistance: Insufficient intracellular drug accumulation, often driven by decreased uptake and ATP-binding cassette (ABC) efflux transporters; Enhanced DNA damage tolerance via upregulated DNA repair pathways; Adaptive signaling and metabolic rewiring that buffers cytotoxic stress; and Protective tumor microenvironmental features, including hypoxia, acidosis, stromal shielding, and immune evasion. Modern coordination complexes, nanoparticles and organometallic agents leverage multiple design elements to bypass transport bottlenecks, overwhelm repair capacity, subvert stress-adaptive signaling, and reshape the tumor niche to restore treatment sensitivity. Note: upward and downward arrows in the name of the cells indicate respectively increase or decrease in their populations.

The aforementioned properties and unique mechanisms of action of metal-based drugs allow them to be effective anticancer compounds. However, cancer cells can evade therapy by forming drug resistance by decreasing effective intracellular drug levels, rerouting metabolic fluxes, reshaping signal transduction, accelerating DNA repair, and exploiting microenvironmental protection. Reduced accumulation spans passive transport limits, diminished influx, enhanced efflux, and the need for precise targeting. Metabolic plasticity—often mitochondrial—supports survival, while specific signaling axes (including those responsive to zinc and arsenic, and pharmacologic p53 re-activators) recalibrate stress responses. Enhanced repair capacity and niche-derived cues further blunt cytotoxicity. We outline how metal-based chemotypes and nanoplatforms address these layers of resistance to restore the therapeutic vulnerability of cancer cells (Fig. 3).

### 5.1 Reducing the Accumulation of the Drug in the Cell:

#### 5.1.1 Passive Transport

Cancer cells can limit intracellular drug exposure by remodeling membrane lipids, thereby affecting both passive permeability and transporter performance. A common adaptation is elevated cholesterol, which reduces membrane fluidity and lowers permeability to polar or charged solutes and many amphipathic drugs. Metal-based therapies are comparatively less dependent on simple diffusion because they often engage carrier-mediated uptake, endocytosis, or specific ion transport pathways. Notably, resistance driven by cholesterol enrichment can also be targeted directly. For example, a fenofibric acid–platinum(IV) conjugate overcame cisplatin resistance by restraining cholesterol accumulation, promoting efflux, and rebalancing lipid metabolism [176].

#### 5.1.2 Drug Influx

Therapeutic efficacy ultimately depends on the ability of the drug to enter cancer cells and reach its intracellular targets. Cancer cells exploit membrane transport systems that either facilitate uptake or promote clearance, thereby shaping sensitivity or resistance [177]. Solute carrier (SLC) transporters mediate facilitated diffusion and secondary active transport of nutrients and xenobiotics, whereas ATP-binding cassette (ABC) transporters export structurally diverse drugs against concentration gradients.

Metal homeostasis intersects these routes. Transferrin receptor 1 (TfR1) internalizes diferric transferrin via clathrin-mediated endocytosis; divalent metal transporter 1 (DMT1/SLC11A2) transports Fe^2+^ and other divalent ions; the high-affinity copper transporter CTR1 (SLC31A1) regulates copper influx [44]; and the copper-transporting P-type ATPases ATP7A and ATP7B mediate copper efflux and intracellular trafficking from the trans-Golgi network [32]. Because copper and iron trafficking are essential for cell viability, they cannot be fully suppressed. Nevertheless, resistance to platinum and copper-mimetic agents frequently involves downregulation of CTR1; intriguingly, copper chelators can reverse this process by reactivating CTR1 and thereby enhancing the uptake of both copper and platinum drugs [178].

Endocytosis offers a complementary, transporter-independent entry route that can be leveraged by metal-based agents. TfR1 not only governs iron uptake but has also been implicated in endocytic internalization of certain ruthenium complexes [179]. In addition, rational surface engineering can trigger receptor-mediated endocytosis, e.g., hyaluronic acid coatings, protease-sensitive peptide modifications, and aptamers targeting nucleolin [180] exemplify strategies that increase uptake while bypassing diffusion limits.

Having considered how metal-based agents enter the cell, we next address how long they can remain inside. In drug-resistant tumors, active export by membrane transporters often dominates intracellular exposure time, regardless of entry route. Many traditional organic chemotherapeutics (e.g., doxorubicin, tyrosine kinase inhibitors) are efficiently recognized by efflux pumps because their hydrophobic or amphipathic profile matches the pumps’ binding pockets [177]. By contrast, metal-based drugs can be engineered to reduce pump recognition, exhaust the energetic basis of efflux, or directly modulate transporter function, while maintaining anticancer activity [181].

#### 5.1.3 Efflux Avoidance, Energy Pressure and Inhibition

One route for prolonged retention is to sidestep recognition by canonical ABC transporters (ABCB1/P-gp, ABCC family, BCRP). Formulations such as Cu(DDC)2 nanoparticles and Elesclomol–Cu nanoparticles display low affinity for P-gp and thereby sustain intracellular levels in multidrug-resistant models [163].

A complementary strategy applies “energy pressure” on pumps: metal-based platforms that trigger excessive intracellular ROS that deplete ATP, thereby attenuating ABC-mediated efflux and consequently increasing drug residency [182]. Some metal-containing agents also act on the transporters themselves as inhibitors. A gold complex (QB1561) partially restores sensitivity to ABCG2 substrates in lung cancer models with ABCG2 overexpression [183]. There were additionally described how gold NPs could inhibit P-glycoprotein (ABCB1) if designed with a particular size [184]. In parallel, nanometal oxides and their ions are used in delivery systems—ZnO/CuO nanoparticles and Cu^2+^/Zn^2+^ salts—have been reported to inhibit P-gp (ABCB1) [185]. Beyond broadly inducible ABC transporters, copper-exporting P-type ATPases (ATP7A/ATP7B) constitute a metal-specific axis of resistance that can bind and sequester both copper and platinum drugs, reducing cytotoxicity. Ion-combination platforms that form TAF-CuET–like species using Fe^3+^ and Cu^2+^ suppress ATP7A/ATP7B and SLC7A11, further engaging ferroptosis and cuproptosis [165]. Such ion-synergistic designs can thus adapt to transporter-driven resistance profiles that hinge on copper handling.

#### 5.1.4 Delivery

While efflux dictates how long a payload stays inside the cell, delivery determines which cells—and which intracellular compartments—are engaged in the first place. Modern delivery strategies for metal-based therapeutics concurrently sharpen selectivity, deepen tissue penetration, and modulate the tumor microenvironment to relax physical and biochemical barriers. Receptor-directed and biomimetic approaches illustrate this convergence: hyaluronic acid for CD44+ populations [182], EpCAM-targeted aptamer nanodrugs [186], amine-rich dendrimers, liposome-polymer nanoparticles [169], and human serum albumin [164]. Biomimetic coatings further extend this toolkit: cancer cell membrane camouflage [187], and multi-responsive platforms with targeting ligands and polydopamine coatings that respond to the tumor microenvironment [188]. Collectively, these delivery innovations provide broadly adaptable strategies that target diverse tumor phenotypes and, critically, enable transit across the blood–brain barrier (BBB) and blood–brain tumor barrier (BBTB).

### 5.2 Altered Metabolism

Chemoresistant cells rewire central metabolism to neutralize oxidative injury, sustain biosynthesis, and stabilize energy supply. Upregulation of the pentose phosphate and one-carbon pathways elevates NADPH and dNTP reserves, quenching drug-induced ROS and attenuating antimetabolites, while expansion of glutathione and thioredoxin networks detoxifies electrophiles and preserves the thiol proteome. In parallel, a shift toward mitochondrial OXPHOS and fatty-acid oxidation sustains ATP and redox balance under mitotic and replication stress, and lipid remodeling limits membrane peroxidation, further diminishing cytotoxic impact. Metal-based agents can invert these protective adaptations by exploiting redox and thiol abundance as activation cues and by directly disabling mitochondrial function. Gold complexes inhibit thioredoxin reductase [183,189], copper and iron complexes deplete glutathione to induce oxidative stress and inhibit glutathione peroxidase 4 (GPX4) [180,182], and mitochondria-targeted Pt(IV)/Ru chemotypes collapse respiration [190]. By converging on OXPHOS, glycolysis, and the Trx axis, these agents also constrain the metabolic plasticity of cancer stem cells, undermining lineage-specific resistances and impairing survival and self-renewal.

### 5.3 Altered Signaling Pathways

Across tumor types, resistant cells rechannel signaling through PI3K–AKT–mTOR, RAS–ERK, Hippo–YAP/TAZ, and STAT3/NF-κB axes, while stress-adaptive nodes (HIFs, NRF2, AMPK, UPR) stabilize redox balance, nutrient acquisition, and lipid anabolism [191,192,193]. Metal-based agents have emerged as multipronged tools to intercept these adaptations.

#### 5.3.1 Co-Targeting Regulated Cell Death and Survival Signaling

Reliance on canonical apoptosis renders tumors vulnerable to defects in p53 or shifts in BCL-2 family balance, as well as compensatory activation of STAT3 and NRF2 programs. Metal complexes that combine orthogonal death programs or pair DNA damage with pathway inhibition can overcome this plasticity. A Pt(IV)–fenofibric acid prodrug augments Bax, engages the NLRP3 inflammasome, activates caspase-1, and cleaves GSDMD—hallmarks of pyroptosis—thereby resensitizing platinum-refractory cells [176]. Synergistic crosstalk of cuproptosis and ferroptosis has likewise been engineered with TAF-CuET, which promotes GPX4 degradation and suppresses SLC7A11, functionally blocking thiol-based defenses and transporter-mediated escape [165]. Rational conjugation can further stack mechanisms of resistance: Pt(IV) fused to an NF-κB inhibitor or EGFR inhibitor elicits DNA damage while constraining compensatory signaling, improving activity in multidrug-resistant settings [194,195].

#### 5.3.2 Restoring p53 Function with Metal-Enabled Strategies

Loss-of-function p53 mutations create a pivotal resistance node. Zinc metallochaperones (e.g., thiosemicarbazones) reinsert Zn^2+^ into zinc-impaired mutants such as R175H, restoring DNA-binding topology and transcriptional output of p53 target genes to resensitize tumors to apoptosis-inducing regimens [196]. For structural mutants such as Y220C, ligands that stabilize mutant-specific surface cavities, and for G245S, covalent binders identified by virtual screening, can allosterically or covalently stabilize active conformations [197]. Arsenic trioxide offers an orthogonal metal-based route by engaging a cryptic allosteric site and promoting refolding of structural mutants, broadening the spectrum of rescue beyond Zn^2+^-dependent mechanisms [198].

#### 5.3.3 Constraining NRF2- and NF-κB-Centered Stress Adaptations

In resistant tumors, NRF2 and NF-κB co-orchestrate antioxidant, inflammatory, and pro-survival programs that buffer cytotoxic stress. Targeting these axes can resensitize cancers to therapy. Cyclometalated ruthenium isoquinoline complexes attenuate PI3K/mTOR signaling, reduce Akt/mTOR phosphorylation, suppress NRF2 target expression, and downregulate MRP1, thereby reversing cisplatin resistance and restoring apoptotic competence [199]. Gold(I) agents provide a complementary route by disrupting the TrxR–Trx system and modulating ERK–MAPK, thereby weakening the NRF2-centered antioxidant shield and promoting apoptosis in platinum-refractory ovarian cancer cells [200]. Nevertheless, Nrf2-associated resistance for metal based compounds is a common challenge and additional inhibitors, along with metal-based therapy, are used [201]. In parallel, NF-κB—frequently activated downstream of Akt/mTOR—induces survival, anti-apoptotic, inflammatory, and DNA-repair programs that blunt cytotoxicity and sustain persistence, including in cancer stem cell compartments [202]. Ru(II)-based complexes with NF-kB inhibition activity demonstrated effectiveness against cancer stem cells (CSCs), which is quite challenging to many target- and chemo-therapies due to their slow proliferation and metabolic activity [203]. 

#### 5.3.4 Targeting BCL-2 Family Dependencies

Shifts toward antiapoptotic BCL-2 and MCL-1 sustain survival under cytotoxic stress. Gold(I) N-heterocyclic carbene complexes downregulate these proteins and restore apoptotic sensitivity in multidrug-resistant leukemia cells, highlighting a route to neutralize mitochondrial checkpoint adaptations [204].

#### 5.3.5 Disarming STAT3-Driven Persistence

Persistent STAT3 activity sustains proliferation, inflammatory crosstalk, and stemness. Metal complexes—including Pt(IV) and half-sandwich Ir(III)—can covalently or coordinatively engage cysteines near STAT3 DNA-binding or SH2-adjacent regions, impeding phosphorylation, dimerization, or DNA binding and producing durable pathway inhibition. Consequent reductions in IL-6/COX-2 signaling reshape the microenvironment and blunt survival cues [205,206]. Tin-based complexes add redox-driven leverage: Sn–hydroxamic acid scaffolds induce oxidative stress, trigger caspase-dependent apoptosis, and suppress STAT3 activity while perturbing the JNK1/MMP axis, collectively resensitizing drug-refractory cells [207].

Together, these findings support that metal-based drugs can be promising substances to target altered cell signaling pathways and stress adaptive nodes of resistant cells.

### 5.4 Targeting Enhanced DNA Repair Mechanisms

Drug resistance driven by enhanced DNA repair is a major barrier to effective cancer therapy, particularly for non-metal-based drugs such as antimetabolites, topoisomerase inhibitors, and targeted agents.

Non-metal-based chemotherapeutics (e.g., 5-fluorouracil, doxorubicin, temozolomide paclitaxel, and targeted kinase inhibitors) often fail due to cancer cells’ upregulation of DNA repair mechanisms: nucleotide excision repair (NER), homologous recombination (HR), base excision repair (BER), and others [208]. For example, resistance to 5-FU and doxorubicin is frequently associated with elevated DNA repair capacity, while PARP inhibitor resistance can arise from restoration of HR function [209].

High copper levels can activate nuclear copper chaperones (e.g., ATOX1), which in turn regulate DNA repair proteins such as MDC1 related to HR/NHEJ mechanisms. Targeting this axis sensitizes resistant tumors to genotoxic drugs [210]. Another effective way to handle enhanced DNA repair is to target mitochondrial DNA (mtDNA) by copper, iridium and ruthenium-based compounds [164,211]. Unlike nuclear DNA, mtDNA lacks a nucleic acid excision repair pathway and histone protection, making it more susceptible to damage. Complexes based on Mn and temozolomide (TMZ) are capable of significantly increasing damage to DNA by also downregulating the expression of MGMT, which repairs DNA lesions after sole temozolomide (TMZ) treatment in glioblastoma. Manganese nanoparticles produce O2 and ROS that downregulate enhanced DNA repair dependent on MGMT, which is crucial in the glioblastoma model [188].

Developing compounds with a multitargeting approach became one of the strategies, especially as the next generation of platinum-based drugs, which were previously highly susceptible to enhanced DNA repair systems. Pt(IV)-based mononitro-naphthalimide conjugate not only induces severe DNA damage higher than that of cisplatin, but also inhibits the repair processes, specifically by down-regulating the RAD51 protein, which is crucial for homologous recombination (HR) and repair of DNA double-strand breaks [212]. Platinum nanozymes induce both DNA platination and oxidative cleavage, disrupting the DNA bending required for NER and preventing repair of platinum-DNA adducts, thus overcoming resistance even in NER-proficient cells [213]. PARP and HDAC inhibitors as ligands in platinum(IV) complexes showed high cytotoxicity in triple-negative breast cancer and glioblastoma cells, disrupting enhanced DNA repair mechanisms, including PARP and BAP, impairing antioxidant defenses, and prolonging cell cycle blockade [208,214].

### 5.5 Tumor Microenvironment

The tumor microenvironment (TME) comprises interdependent cellular (reprogrammed fibroblasts and immune cells) and acellular components (hypoxia, pH, and extracellular matrix (ECM)) that collectively limit the efficacy of cytotoxic chemotherapies, targeted small-molecule inhibitors, and monoclonal antibodies. Several hallmark features of the TME converge to reduce drug delivery, alter drug metabolism, and induce adaptive resistance programs in cancer cells. On the contrary, metal compounds are able to effectively circumvent some obstacles associated with the tumor microenvironment.

Hypoxia arises from disorganized, leaky, and poorly perfused vessels, promoting genetic instability and pro-angiogenic signaling that further lead to vascular abnormality. Hypoxia reprograms tumor metabolism toward glycolysis and redox plasticity, increases glutathione and NADPH buffering, upregulates drug-efflux transporters (e.g., ABC family), and fosters epithelial–mesenchymal transition and stem-like states that better tolerate DNA damage and apoptotic signals. These changes blunt the activity of DNA-damaging chemotherapies, many kinase inhibitors, and antibody therapeutics that rely on adequate perfusion for delivery. Metal-based compounds are able to withstand hypoxia in several ways. Firstly, metal peroxide nanoparticles (e.g., manganese TMZ@HMnO2@PDA-PEG-RAP12 and calcium peroxides) react with the acidic tumor environment to generate oxygen, alleviating hypoxia and sensitizing tumors to therapy. This oxygen release can restore the efficacy of oxygen-dependent treatments and reduce HIF-1α-mediated resistance pathways [188]. Secondly, the hypoxic environment can be a selective target for some metal-based drugs (e.g., HA-CuMOF@DOX and CuGI@CM) [182,187]. Thirdly, the hypoxia can be deregulated through the inhibition of cell signaling, PI3K/AKT, STAT-3.

Cancer-associated fibroblasts (CAFs) deposit and crosslink collagen and hyaluronan, stiffening and densifying the ECM, compressing vessels, and establishing steep gradients of oxygen, pH, and nutrients. The resulting elevated interstitial fluid pressure and tortuous diffusion paths impede penetration of small molecules and especially large biologics (mAbs, ADCs). CAF-derived cytokines and growth factors (e.g., IL-6, TGF-β, HGF) provide parallel survival cues that reduce dependence on the intended drug target and promote reversible drug tolerance. Au@Ag and AgNPs inhibit the cancer-promoting activity of CAFs by modulating their secretory profiles and altering their interaction with cancer cells. They also reduce the expression of genes related to cancer invasion and metastasis, such as secreted phosphoprotein 1 (Spp1, which encodes osteopontin), pleiotrophin (Ptn), thrombospondin 2 (Thbs2), and ADAM metallopeptidase with thrombospondin type 1 motif 5 (Adamts5) [215]. Disulfiram/copper (DSF/Cu) induces apoptosis and necrosis in both cancer cells and α-SMA-positive CAFs. It downregulates CAF activation markers (α-SMA, FAP-α), functionally inactivating CAFs and reducing their tumor-supportive activity [216].

Tumor-associated macrophages, neutrophils, and myeloid-derived suppressor cells create an immune-privileged niche by supplying growth and survival factors, scavenging reactive oxygen species, remodeling the ECM, and expressing checkpoint ligands that blunt cytotoxic T-cell activity. Therapy itself can reinforce these programs by inducing senescence-associated secretory phenotypes and wound-healing signals. Such myeloid-driven circuits can limit the efficacy of mAbs that require Fc-mediated effector functions, attenuate immunogenic cell death from chemotherapy, and provide bypass signaling that undermines targeted inhibitors. Drugs based on iron and copper, or containing both metals simultaneously, have shown the ability to prevent low immunogenicity in tumor environments by inducing ferroptosis and cuproptosis. These drugs can repolarize tumor-associated macrophages (TAMs) from the M2 phenotype to the M1 phenotype, increase intratumoral infiltration of CD8^+^ cytotoxic T lymphocytes and natural killer cells, which has an antitumor effect [163,164]. Copper-deposited metal-phenolic networks (TAF-CuET), Manganese Nanoparticles and Pt-conjugates are also capable of reducing the fraction of regulatory T cells (Tregs) and myeloid-derived suppressor cells (MDSCs), and activating cytotoxic T lymphocytes [165,217]. 

Extracellular vesicle transport from resistant cancer cell lines to susceptible ones was reported as a problem for TKI treatment. Transported SLC-transporters (SLC1A5 and SLC25A5) activated metabolism change to OXPHOS in cancer cells and immunosuppression of tumour microenvironment (polarization of macrophages to M2 and inhibition of dendritic cell function) [218]. This description creates good initial conditions for further research regarding the use of metal-based compounds.

### 5.6 Functionalized Metal-Based Nanoparticles as a Distinct Type of Therapeutic Modality

Metal nanoparticles (MNPs), as briefly mentioned above, represent one of the most famous fast-developing and frequently used forms of metal-based therapeutics. In previous sections, we were mostly focused on the pure mono-metal nanoformulations, while there are numerous attempts to create complex nanosystems with interesting properties and biological activity. This vast field of research is beyond the scale of this article so here we mention only several important trends that we consider necessary additional attention in the context of this review.

The first trend is the creation of multi-metal NPs, that can be in a form of polymetallic alloy or a more complex multilayered structure. These formulations can combine unique properties of several metals. For example, multifunctional MNPs with a combination of iron and noble metals (silver, gold, or platinum) acquire magnetic properties with simultaneously higher stability, lower reactivity, and tuned optical properties (plasmon resonance), which enables multimodal imaging [219]. Gold-copper alloys possess enhanced catalytic and photo-optic properties [220], which found their use in cancer theranostics [221].

The second major tendency is the creation of sophisticated organic coatings and the application of drug payloads for precise targeting. This process radically changes the physico-chemical properties of NPs as well as their biological behaviour and activity. MNPs can be modified with small organic molecules, polyorganic shells, biomolecules (nucleic acids or proteins), and other types of coagulants [222]. Each modification brings a new feature to the nanosystem, for example, polyethylene glycol (PEG) is used for shielding active metal core from the surroundings [223], folic acid targets NPs toward cancer cells overexpressing folate receptors [224], hyaluronic acid and other polymers for drug delivery [225], chitosan improves NPs stability and delivery capacity [226]. Moreover, important modifiers are antibodies and polypeptides, which can be designed to target specific cells or suppress specific molecular targets [227]. Although these biomodifications really widen the spectrum of possible applications of NPs, the concern over immunogenicity and toxicity still remains.

Overall, we can expect major breakthroughs in the field of functionalized metal-based nanoparticles and approvals from the FDA for their use in many fields, including cancer diagnosis and treatment. Current solutions gravitate around various types of coatings and shells with simultaneous experiments with a metallic core, which combined make the number of possible solutions and formulations almost infinite.

To illustrate the translational potential of metal-based nanoparticles in overcoming MDR, Table 4 summarizes representative *in vitro* and *in vivo* studies. These examples highlight how MNPs can bypass efflux pumps, modulate the tumor microenvironment, induce novel cell death pathways, and synergize with existing therapies to resensitize resistant cancers.

**Table 4:** Representative *in vitro* and *in vivo* studies of metal nanoparticles (MNPs) for overcoming multidrug resistance in cancer.

Metal	MNPs Formulation	Cancer Model (Cell Line/Animal)	Dosage/Concentration	Key Findings, Mechanism	Ref.
*Au*	Au NPs (PEGylated, ~20 nm)	MCF-7/ADR cells (*in vitro*)	10–50 μg/mL	Bypassed P-gp efflux, induced ROS-mediated apoptosis, restored doxorubicin sensitivity	[184]
*Au*	Au nanorods (anti-EGFR conjugated)	A549 xenograft (*in vivo*)	5 mg/kg, i.v.	Enhanced tumor accumulation, combined with PTT + chemotherapy, reduced tumor volume by 80%.	[156]
*Fe*	Ferumoxytol (SPIONs)	4T1 murine breast cancer (*in vivo*)	5 mg Fe/kg, i.v.	Repolarized TAMs to M1 phenotype, enhanced CD8^+^ T-cell infiltration, suppressed tumor growth.	[228]
*Cu*	CuET NPs (disulfiram-derived)	PC-3 prostate cancer (*in vitro*, *in vivo*)	10 μM (*in vitro*); 5 mg/kg (*in vivo*)	Inhibited ATP7A/B, induced cuproptosis, overcame cisplatin resistance in xenografts.	[229]
*Mn*	MnO_2_ NPs + temozolomide	U87MG glioblastoma (*in vivo*)	2 mg Mn/kg, i.v.	Relieved hypoxia, downregulated MGMT, enhanced TMZ efficacy in resistant GBM.	[188]
*Pt*	Pt nanozymes (Pt@ZIF-8)	A2780 cisplatin-resistant ovarian cancer (*in vitro*)	20 μM Pt equivalent	Catalyzed ROS generation, disrupted DNA repair (nucleotide excision repair), synergized with PARP inhibitors.	[213]
*Ag*	AgNPs (triangular, ~50 nm)	Panc-1 pancreatic cancer (*in vitro*)	25 μg/mL	Induced mitochondrial dysfunction, inhibited CAF activation, reduced invasion markers.	[215]
*Cu/Fe*	TAF-CuET metal-phenolic networks	4T1 TNBC model (*in vivo*)	10 mg/kg, i.v.	Dual cuproptosis/ferroptosis, reprogrammed TME, enhanced anti-PD-1 response.	[165]

Note: CuET, copper(II) diethyldithiocarbamate; GBM, glioblastoma; MGMT, O6-methylguanine-DNA methyltransferase; NPs, nanoparticles; PARP, poly (ADP-ribose) polymerase; PEG, polyethylene glycol; SPIONs, superparamagnetic iron oxide nanoparticles; TMZ, temozolomide; TNBC, triple-negative breast cancer; ZIF, zinc imidazolate.

Some modern concepts of future drug therapy propose combining several therapeutic modalities into one system in order to address different aspects of tumor progression and the immunosuppressive tumor microenvironment (TME). One study proposes a therapy that includes photothermal, chemodynamic therapy, and immunomodulation caused by copper ions (Cu^2^^+^), in combination with an anti-PD-L1 antibody and S-nitrosoglutathione (GSNO), for the regulation of cancer-associated fibroblasts on a hydrogel platform that controls the release of compounds [230].

## 6 Limitations of Metal-Based Formulations

Despite a significant number of advantages over metal-free therapy, it is necessary to note a number of disadvantages, limitations, and even dangers associated with metal-based therapy. First of all, systemic toxicity remains a major barrier for metal-based anticancer agents. Classic platinum drugs (cisplatin, oxaliplatin, etc.) cause severe dose-limiting toxicities—most notably nephrotoxicity, neurotoxicity and ototoxicity—that curtail therapeutic dosing [231]. Moreover, long-term accumulation of platinum-based drugs (e.g., oxaliplatin) in cancer-associated fibroblasts CAFs can paradoxically promote cancer progression and resistance. CAFs retain platinum, which intensifies TGF-β signaling and secretion of pro-tumorigenic factors, supporting tumor aggressiveness and therapy resistance [232]. Similarly, other redox-active metal drugs induce off-target oxidative damage to normal tissues, further narrowing the therapeutic window [233]. Transition-metal complexes that catalytically generate reactive oxygen species can kill cancer cells but also deplete antioxidants (e.g., oxidizing GSH) and harm normal cells [234]. In sum, the broad reactivity of most metal drugs produces collateral damage (e.g., DNA and membrane oxidation) in normal organs at doses needed to affect tumors, severely limiting safe dosing.

Metal agents also face daunting biodistribution and bioaccumulation challenges. Nanoparticles or long-lived metal complexes often fail to achieve selective tumor accumulation. In practice, inorganic carriers are rapidly sequestered by the mononuclear phagocyte system, leading to high uptake in the liver, spleen and other organs rather than tumors [235]. For instance, studies of silver nanoparticle biodistribution show the majority of the dose deposited in spleen and liver (41.5% and 24.5%, respectively, at 24 h) with negligible tumor delivery [154]. Persistent metal complexes similarly tend to accumulate in off-target tissues over time, raising long-term toxicity. Moreover, tumor models in mice often vastly overestimate the “enhanced permeation and retention” effect; human tumors have more variable perfusion and stroma, meaning nanoparticle uptake seen preclinically often does not translate to patients [236].

Off-target reactivity and narrow therapeutic indices further compound these issues. Many metallodrugs depend on general redox or ligand-substitution chemistry that cannot be fully controlled *in vivo*, so they react with abundant biological nucleophiles (for example, cellular thiols such as glutathione or biomacromolecules like nucleic acids) as readily as with tumor targets. This nonspecific activation produces a steep dose–response: modest dose escalations often sharply increase systemic toxicity with only limited gains in tumor killing, because circulating reductants activate prodrugs or metal catalysts before they reach the tumor [237,238]. For example, Pt(IV) prodrugs must be reduced to Pt(II) to bind DNA (and Cu(II) to Cu(I) in many copper-based systems); however, ubiquitous reductants such as glutathione or ascorbate can prematurely reduce and thereby activate these species in healthy tissues, leading to systemic toxicity and loss of tumor selectivity. Likewise, redox-cycling metal centers and catalytic metal complexes can promote wide-ranging ROS formation (via Fenton-type or redox cycles) and deplete thiol buffers, causing off-target oxidative damage in non-tumor organs unless activation is tightly confined to the tumor microenvironment [239].

Finally, practical and translational issues impose additional barriers. Complex metal formulations (e.g., nanoparticle-encapsulated or multimodal chelates) are notoriously difficult to manufacture and formulate reproducibly. Large-scale synthesis must ensure precise control of size, composition and purity—a challenging requirement for inorganic assemblies—yet batch-to-batch variability is common. Regulatory agencies have limited experience with novel metallodrugs, so the path to approval is encumbered by extensive safety requirements and impact on the body, offspring, environment. Indeed, recent reviews note that “scalability, manufacturing reproducibility, and regulatory approval remain significant hurdles” for metal-containing nano-therapeutics [240,241]. After all, the leap from preclinical rodent models to human therapy is a daunting challenge. Many promising metal-drug effects in mice (due to conventional tumor models, mouse metal metabolism, or idealized perfusion) simply do not replicate in patients. Human tumors often have poorer vascular access and different redox environments than mouse xenografts, and there are still few clinical biomarkers to guide patient selection for metal-based therapies [242]. Taken together, these factors—extreme toxicity, poor targeting/biodistribution, unwieldy manufacturing, and species differences—create a formidable translational gap for metal-based anticancer agents that should be addressed in future studies.

## 7 Conclusion and Future Perspectives

Metal-based drugs and therapeutic approaches have garnered significant attention in cancer therapy, presenting both compelling advantages and distinct challenges. Starting with cisplatin, a paradigm-shifting agent that damages DNA through crosslinking, the field has evolved to include sophisticated formulations with diverse and multimodal mechanisms of action. These mechanisms now extend far beyond simple DNA intercalation to encompass the induction of novel regulated cell death pathways like ferroptosis and cuproptosis, modulation of the tumor immune microenvironment, unique redox-driven signaling disruption, and advanced delivery strategies.

The primary advantage of metal-based therapeutics, as detailed in this review, lies in their inherent chemical versatility and multimodal activity. This allows them to bypass classical resistance mechanisms—such as drug efflux pumps, enhanced DNA repair, and metabolic rewiring—which frequently limit the efficacy of conventional organic chemotherapeutics. The ability of metal complexes to engage in redox cycling, coordinate with diverse biological targets, and be engineered into advanced nanoformulations positions them as uniquely capable of inducing multimodal stress responses that resistant cells cannot easily evade. Furthermore, the frequent dysregulation of metal homeostasis in cancers provides a therapeutic window for selective targeting, exploiting, for instance, the overexpression of critical transporters and factors, like transferrin, ion channels or specific metal transporters.

However, these advantages are counterbalanced by significant disadvantages and translational hurdles. Systemic toxicity—notably nephrotoxicity, neurotoxicity, and myelosuppression associated with platinum drugs—remains a major clinical limitation. The redox activity of many metal agents can cause off-target oxidative damage, narrowing the therapeutic window. Challenges in biodistribution, such as nonspecific accumulation in the liver and spleen, and poor tumor penetration, often undermine the efficacy of metal-based nanoparticles. Moreover, complex metal formulations face practical challenges in scalable manufacturing, reproducibility, and regulatory approval due to their inorganic nature and potential long-term bioaccumulation.

The convergence of these aspects leads to a clear conclusion: there is a compelling necessity for extensive and strategic research efforts dedicated to advancing metal-based therapeutics. The future of this field lies not merely in the incremental creation of new metal-organic complexes, but in the rational design of combinatorial and precision approaches that integrate knowledge from chemistry, biology, and materials science. Next steps of development must be focused on enhancing tumor selectivity through precision targeting, leveraging biomarkers of metal dysregulation and functionalizing complexes (like LOX, COX, SOD) with specific moieties to mitigate the systemic toxicity that has historically been a limitation.

The greatest potential may lie in fully exploiting synergistic multimodal therapies, intentionally combining metallodrugs with immunotherapy, targeted agents, and radiotherapy to create potent, multi-pronged attacks on resistant cancers. Advanced, programmable platforms such as metal-organic frameworks (MOFs) epitomize this integrative philosophy, capable of merging high-fidelity targeting, multi-agent delivery, intrinsic catalytic activity, and immune modulation within a single, tunable architecture. Concurrently, the exploration of underutilized metals and their unique mechanisms, such as specific immune pathway activation or mitochondrial targeting, will be essential to uncover new vulnerabilities. Ultimately, by thoughtfully addressing the challenges of toxicity and manufacturing, and by embracing these innovative strategies, the next generation of metal-based therapies holds exceptional promise for transitioning from being drugs of last resort to becoming central components of modern, personalized cancer treatment regimens designed to overcome the formidable challenge of drug resistance.

## Data Availability

Data sharing is not applicable (only appropriate if no new data is generated or the article describes entirely theoretical research).

## Abbreviations

ABC	ATP-binding cassette
ATP7A/B	copper-transporting ATPase 7A/B
CAFs	cancer-associated fibroblasts
CAR-T	chimeric antigen receptors
CaX NPs	calcium-based nanoparticles
CD19	cluster of differentiation 19, B-lymphocyte antigen
CDT	chemodynamic therapy
CNTs	carbon nanotubes
CTLA-4	cytotoxic T-lymphocyte-associated protein 4
dNTP	deoxyribonucleotide triphosphates
ECM	extracellular matrix
EGFR	epidermal growth factor receptor
ENSA	endosulfine alpha
HER2	human epidermal growth factor receptor 2
ICD	immunogenic cell death
i.v.	intravenous
MDR	multi-drug resistance
NADPH	Nicotinamide adenine dinucleotide phosphate
NPs	nanoparticles
OXPHOS	oxidative phosphorylation
PARP	poly(ADP-ribose) polymerase
PD-1	the programmed cell death protein 1
PEG	polyethylene glycol
PDT	photodynamic therapy
PTT	photothermal therapy
RedOx	reduction-oxidation
ROS	reactive oxygen species
TAMs	tumor-associated macrophages
TKI	tyrosine-kinase inhibitors
TME	tumor microenvironment
